# Are Aflatoxin Residues in Chicken Products a Real or Perceived Human Dietary Risk?

**DOI:** 10.3390/toxins17040179

**Published:** 2025-04-04

**Authors:** Madalitso Chelenga, Limbikani Matumba, Muloongo C. Sitali, Bertha Kachala, Verson Nambuzi, Merning Mwenifumbo, Aggrey Pemba Gama, Mulunda Mwanza, Maurice Monjerezi, John F. Leslie

**Affiliations:** 1 Department of Veterinary Clinical Studies, Faculty of Veterinary Medicine, Lilongwe University of Agriculture and Natural Resources (LUANAR), Lilongwe P.O. Box 219, Malawi; versonnambuzi@gmail.com (V.N.); mmwenifumbo@luanar.ac.mw (M.M.); 2 FoodPlus Research Group, Faculty of Life Sciences Natural Resources, LUANAR (NRC Campus), Lilongwe P.O. Box 143, Malawi; bkachala@luanar.ac.mw; 3 Department of Biomedical Sciences, School of Veterinary Medicine, The University of Zambia, Lusaka P.O. Box 32379, Zambia; muloongosit2013@gmail.com; 4 Food Science and Technology Department, Faculty of Food and Human Sciences, LUANAR (Bunda campus), Lilongwe P.O. Box 219, Malawi; agama@luanar.ac.mw; 5 Department of Animal Health, Faculty of Natural and Agricultural Sciences, North-West University, Private Bag X2046, Mmabatho 2745, South Africa; mulunda.mwanza@nwu.ac.za; 6 Centre for Resilient Agri-Food Systems (CRAFS), University of Malawi, Zomba P.O. Box 280, Malawi; mmonjerezi@unima.ac.mw; 7 Department of Plant Pathology, Throckmorton Plant Sciences Center, Kansas State University, 1712 Claflin Rd., Manhattan, KS 66506, USA; jfl@ksu.edu

**Keywords:** animal feed, edible tissue, food safety, maximum tolerable level, mycotoxins, poultry

## Abstract

Aflatoxin is a health threat to humans and domesticated animals. Chickens are often fed aflatoxin-contaminated grain and may retain some toxins in muscle, eggs, and other tissues. A critical food safety question is whether tissues from contaminated birds pose a threat to the humans that consume them. We evaluated literature published from 1984 to 2023 to determine the level of aflatoxin residues retained in chicken eggs, muscles, livers, gizzards, and hearts. In the studies evaluated (*n* = 33), ~8100 chickens in 334 trials were fed feed contaminated with 0.1–6400 µg/kg of aflatoxins for 7–180 days. There was a positive correlation between the level of feed contamination and residual aflatoxin concentrations (*r*^2^ = 0.18, *p* < 0.05), but <1% of the aflatoxin in the feed carried over to edible broiler tissues. Only 0.6% of the trials reported >20 µg/kg of aflatoxin in the tissues, primarily in the muscle tissue, when the chickens were fed feed contaminated with >300 µg/kg of aflatoxins, which is above the US FDA maximum tolerable limit for components of poultry feeds. These composite results suggest a relatively low risk to public health from consuming chickens fed contaminated feed and a relatively high aflatoxin elimination mechanism in chickens that consume feed containing >300 µg/kg of aflatoxins. The data are consistent with chickens fed feed containing up to 500 µg/kg of aflatoxin being allowed in the human food chain without posing a significant health hazard. In reality, the maximum level of aflatoxin allowed in chicken feed will probably be limited by how much the birds can tolerate and still grow at a suitable rate without deformities rather than the risk that processed birds could present to human health. As chickens effectively act as an absorptive buffer for aflatoxin in contaminated feed, we expect that a contamination level that is acceptable for chicken growth performance is likely to be less than the amount that keeps chicken products safe for human consumption.

## 1. Introduction

Health and growth impairment of turkeys consuming contaminated grain [1] led to the original characterization of aflatoxins and their identification as a significant threat to the health of humans and domesticated animals. Extensive efforts have been made to determine safe feed exposure levels and develop regulatory guidelines for these toxic, thermostable secondary metabolites that cause aflatoxicosis, cancer, and liver failure in humans and animals. The primary focus has been on contaminated grains and plant products; however, aflatoxin residues have been identified in animal tissues, and animal products also could be human health threats [2]. Human exposure to aflatoxins can lead to a myriad of health problems including, but not limited to, carcinogenesis, reproductive dysfunction [3], and impairment of the immune system [4]. Humans are primarily exposed to aflatoxins, most commonly aflatoxin B_1_ (AFB_1_), via the consumption of contaminated cereals (maize, sorghum, rice, wheat) and oilseeds (peanut, sunflower, soybean, cotton) [5]. These same agricultural commodities are also fed to animals directly or as ingredients in formulated feeds. Aflatoxins can carry over to animal products, e.g., milk, eggs, and meat, with contamination of chicken products of particular importance in developing countries. The efficiency of the transfer of aflatoxin from feed to consumable chicken products is a critical factor in determining the suitability of these products for human consumption.

Our objective in this review was to consolidate available data on the efficiency of aflatoxin transfer from feed to chicken products. Our working hypotheses were that aflatoxin contamination in chicken tissues depends (i) on the level of aflatoxin in the feed, (ii) the length of time the contaminated feed was consumed, and (iii) that chickens consuming feed contaminated at levels above that allowed in the feed would result in chicken tissues that pose a hazard to human health. Our results advance the field by identifying that <1% of the aflatoxins in chicken feed are retained in chicken tissues that are consumed by humans.

## 2. Results

### 2.1. Characteristics of the Included Studies and the Aflatoxin Exposure Profiles in the Evaluated Chickens

**Figure 1:**
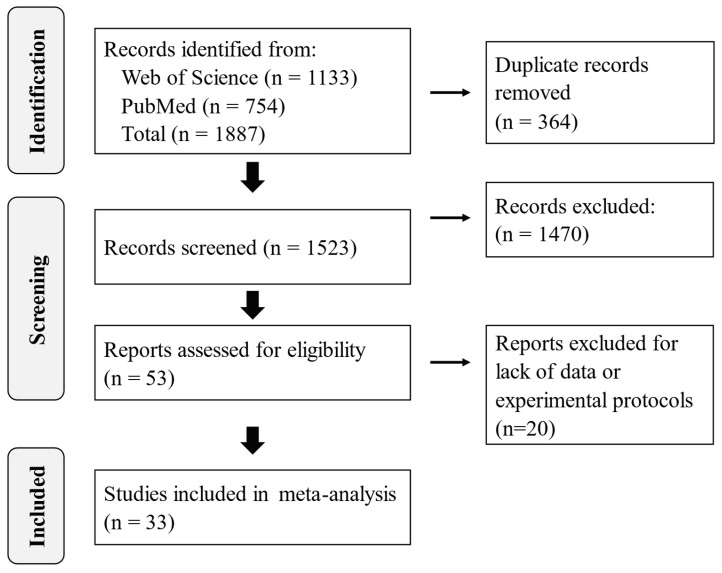
PRISMA flow diagram.

Thirty-three studies conducted in different parts of the world were selected for this study (Figure 1, Table 1). These studies evaluated over 8100 chickens from 342 trials. Four of the 33 studies contained eight trials that did not report the levels of aflatoxins in the feed [6,7,8,9] and were subsequently excluded from further analysis. In the remaining 334 trials, an average of 1,700 µg of aflatoxin/kg of contaminated feed (range = 0.1–6400 µg/kg, median = 1680) was fed to chickens for 7–180 days. About two-thirds of the trials in these studies introduced aflatoxin by incorporating fungal-colonized grains on which aflatoxins had been produced into the chicken feed. In the remaining one-third, aflatoxin was introduced through artificial spiking with one or more of aflatoxins B_1_, B_2_, G_1_, or G_2_. One study did not report the source of the aflatoxins used in the experiments.

### 2.2. Aflatoxin Carryover to Various Chicken Tissues

**Figure 2:**
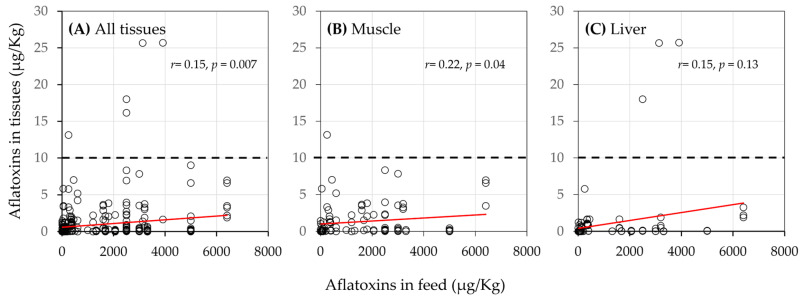
Relationship between the concentration of aflatoxin in chicken feed and aflatoxin residue in (**A**) all chicken tissues, (**B**) muscle, and (**C**) liver. The red lines are the line of best fit. The dotted lines indicate the global median limit of 10 µg/kg for total aflatoxins.

**Figure 3:**
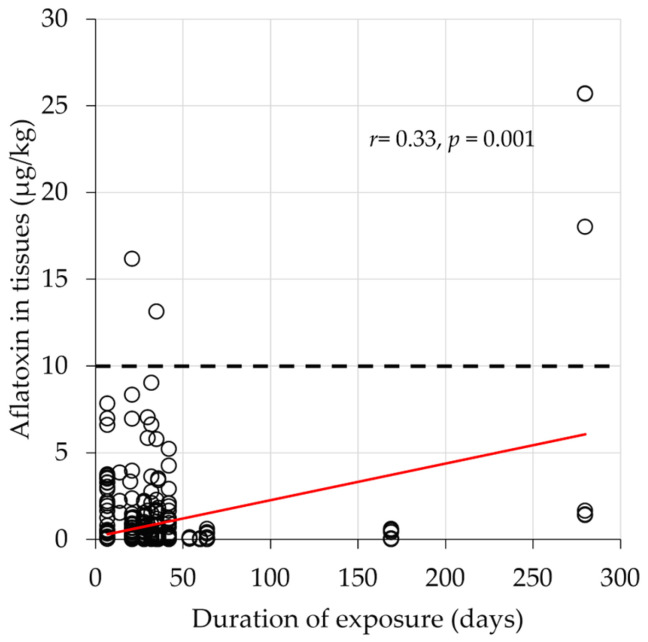
Relationship between the duration of exposure and the accumulation of aflatoxin residues in various tissues derived from chickens fed with aflatoxin-contaminated feed. The red line is the line of best fit. The dotted line indicates the global median limit of 10 µg/kg for total aflatoxins.

Generally, the concentration of aflatoxin residues in chicken tissues positively correlated with the concentration in the chicken feeds (*r^2^* = 0.15, *p* = 0.007, Figure 2A). There also was a significant association between aflatoxin accumulation in a tissue and the duration of exposure to aflatoxin (*r^2^* = 0.33, *p* < 0.001, Figure 3). The *r*^2^ values reported suggest that 15% of the variation reported for the aflatoxin retained in the tissue was due to the aflatoxin concentration in the feed and that 33% of the variation observed was due to length of exposure [39]. Nonetheless, the average aflatoxin tissue contamination across all trials and tissues was ~0.5 µg/kg, with the highest average in the gizzards at 1.4 µg/kg of aflatoxins. Thus, we hypothesize that chickens have strong aflatoxin-buffering capability.

When chickens were fed feed contaminated with <100 µg/kg of aflatoxins (*n* = 80), 91% of the trials found aflatoxin residue levels <1 µg/kg of the aflatoxin in tissues, and none had a residue level higher than 10 µg/kg (Table 2). If the aflatoxin levels in the feed were between 100 and 300 µg/kg, then 77% of the trials reported <1 µg/kg of aflatoxin in the tissues, and none reported >20 µg/kg aflatoxin residue. Most (*n* = 219) of the studies fed chickens feed contaminated with >300 µg/kg of aflatoxins, which exceeds the FDA’s allowable limit for aflatoxin in poultry feed. Seventy-four percent of the trials reported <1 µg/kg of aflatoxin in the tissue, an additional 25% reported <20 µg/kg, and only two trials reported residual levels above the FDA’s current regulatory limit of 20 µg/kg [7]. The median amount of aflatoxin residue in all of the tissues was significantly associated (*r*^2^ = 0.15, *p* = 0.007) with the amount of aflatoxin present in the feed.

#### 2.2.1. Aflatoxin Accumulation in Chicken Muscle

Of the 91 trials reporting aflatoxin levels in feed and residue accumulation in chicken muscles (Table 2), only 38 used feed contaminated with <300 µg/kg of aflatoxin. Amongst these 38 trials, 34 had aflatoxin residues that were <1 µg/kg, and the highest value any of the 4 remaining trials had was <10 µg/kg of aflatoxins. The remaining 53 trials with data for chicken muscle used >300 µg/kg aflatoxin contamination in the feed. Two trials had chickens with residues >20 µg/kg and 40 had chickens with residues that were <1 µg/kg of aflatoxins.

#### 2.2.2. Aflatoxin Accumulation in Chicken Liver

Of the 103 trials reporting aflatoxin levels in feed and subsequent residues in the liver (Table 2), 24 used feed contaminated with <100 µg/kg of aflatoxins with 88% having <1 µg/kg of aflatoxins in the liver and none having >10 µg/kg of aflatoxin residue. In the 12 trials with the feed contaminated with 100–300 µg/kg of aflatoxins, 8 trials reported <1 µg/kg of aflatoxin residue and the other 4 all reported <20 µg/kg residue in the liver. Again, most of the trials (*n* = 67) used >300 µg/kg of aflatoxin in the contaminated feed with 57% reporting <1 µg/kg of aflatoxin residue and the remaining 43% all below 10 µg/kg of aflatoxin residue in the liver (Table 2). None of these trials reported >10 µg/kg of aflatoxin residues in the liver. The median amount of aflatoxin residue in the liver was not significantly associated (*r*^2^ = 0.15, *p* = 0.013) with the amount of aflatoxin present in the feed.

#### 2.2.3. Aflatoxin Accumulation in Chicken Eggs

Fifty-eight trials (Table 2) reported aflatoxin in chicken feed and in eggs laid by chickens that consumed the contaminated feed. If the feed was contaminated with <300 µg/kg of aflatoxin, then the aflatoxin residue was <1 µg/kg in all of the eggs. In the 41 trials that used feed contaminated with >300 µg/kg of aflatoxin, 38 of the trials reported aflatoxin residues <1 µg/kg in the eggs. None of these trials reported aflatoxin residues of >5 µg/kg in the eggs.

#### 2.2.4. Aflatoxin Accumulation in Chicken Kidney

Thirty-five trials reported aflatoxin levels in feed and subsequent residue accumulation in chicken kidneys (Table 2). Twenty-four of the trials used feed contaminated with >300 µg/kg of aflatoxin. Twenty of these trials reported aflatoxin residues <1 µg/kg and none of the trials had kidneys with residues that were >20 µg/kg. If the feed was contaminated with <300 µg/kg of aflatoxin, then 7/11 trials had <1 µg/kg of aflatoxin in the kidney and the remaining 4 trials all had aflatoxin residues that were <5 µg/kg.

**Table 2:** Total aflatoxin concentration in the feed and subsequent concentration of the residues in various edible tissues.

Tissue (Total Number of Trials)	Aflatoxin in Feeds		Number of Trials (%) with Different Ranges of Aflatoxin Concentration (µg/kg) in Chicken Tissues				
	Concentration (µg/kg)	Number of Trials	<1	1–5	5–10	10–20	>20
Total (334)	0.1−100	80	73 (91)	6 (8)	1 (1)		
	100−300	35	27 (77)	6 (17)	1 (3)	1 (3)	
	>300	219	161 (74)	45 (20)	9 (4)	2 (1)	2 (1)
AFB_1_ only (192)	0.1−100	52	48 (92)	3 (5)	1 (2)	1 (2)	
	100−300	25	17 (68)	6 (24)	2 (8)		
	>300	115	65 (56)	38 (33)	8 (7)	2 (2)	2 (2)
Liver (103)	0.1−100	24	21 (88)	2 (8)	1 (4)		
	100−300	12	8 (67)	3 (25)		1 (8)	
	>300	67	38 (57)	23 (34)	6 (9)		
Muscle (91)	0.1−100	21	20 (95)	1 (5)			
	100−300	17	14 (82)	2 (12)	1 (6)		
	>300	53	40 (75)	10 (19)		1(2)	2 (4)
Eggs (58)	0.1−100	13	13 (100)				
	100−300	4	4 (100)				
	>300	41	38 (93)	3 (7)			
Kidneys (35)	0.1−100	10	7 (70)	3 (30)			
	100−300	1		1 (100)			
	>300	24	20 (84)	2 (8)	1 (4)	1 (4)	
Gizzards (25)	100−300	1	1 (100)				
	>300	24	15 (63)	7 (29)	2 (8)		
Skin (8)	0.1−100	8	8 (100)				
Heart (9)	0.1−100	4	4 (100)				
	>300	5	5 (100)				
Spleen (5)	>300	5	5 (100)				

#### 2.2.5. Aflatoxin Accumulation in Chicken Gizzards

Twenty-five trials reported aflatoxin levels in chicken feed and residues in the chicken gizzards (Table 2). All but one trial used feed contaminated with >300 µg/kg. Sixteen of the trials resulted in aflatoxin residues <1 µg/kg and the remaining nine all had residues <10 µg/kg of aflatoxin in the gizzards.

#### 2.2.6. Aflatoxin Accumulation in Chicken Skin, Heart, and Spleen

A few trials evaluated aflatoxin levels in feed and residues in the chicken skin, heart, and spleen (Table 2). In all of these trials, the aflatoxin residues in the tissues were all < 1 µg/kg regardless of the concentration of aflatoxins in the feed.

### 2.3. Aflatoxin Buffering Capacity by Tissue

**Figure 4:**
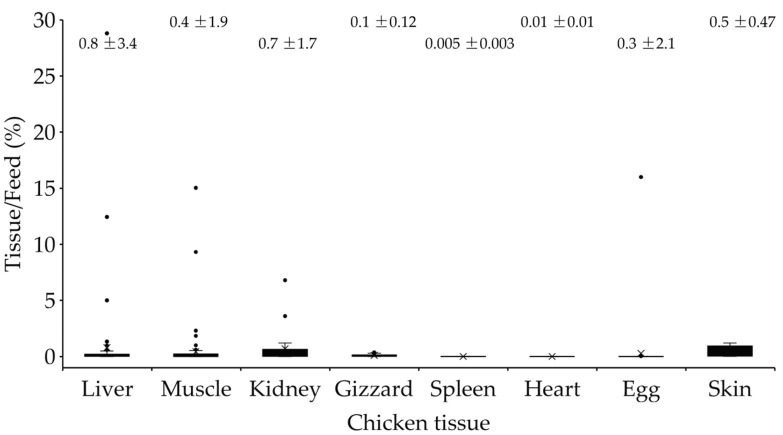
Aflatoxin buffering ability of chickens determined by the percent fold change (tissue: feed ratio) of aflatoxin concentration found in contaminated chicken feeds relative to the concentration of aflatoxin residues accumulated in the various tissues. Numbers above each column are mean ± standard deviation. Solid blocks in each column represent the 25th to 75th percentile range. “X” indicated the median value observed. Bullets are values > 75th percentile.

Aflatoxin buffering capacity varied by chicken tissue (Figure 4). Metabolically active organs, such as the liver and kidneys, retained higher levels of aflatoxin and exhibited weaker buffering, while muscle, eggs, and skin exhibited higher buffering capacity. The liver, a primary detoxification organ, had limited buffering capacity as 43% of the trials detected aflatoxin residues >1 µg/kg when feed contamination exceeded 300 µg/kg. Kidneys and gizzards also had moderate buffering capacity, retaining aflatoxins in 17–37% of the trials conducted with feed contaminated with >300 µg/kg of aflatoxins. In contrast, muscle and eggs displayed strong buffering capacity, with no significant aflatoxin residues detected in 76–100% of the relevant trials. Tissues such as skin, heart, and spleen exhibited the highest buffering capacity, with no measurable aflatoxin residue detected in any of the trials.

### 2.4. Effect of Aflatoxin Source Used to Contaminate Feed

**Figure 5:**
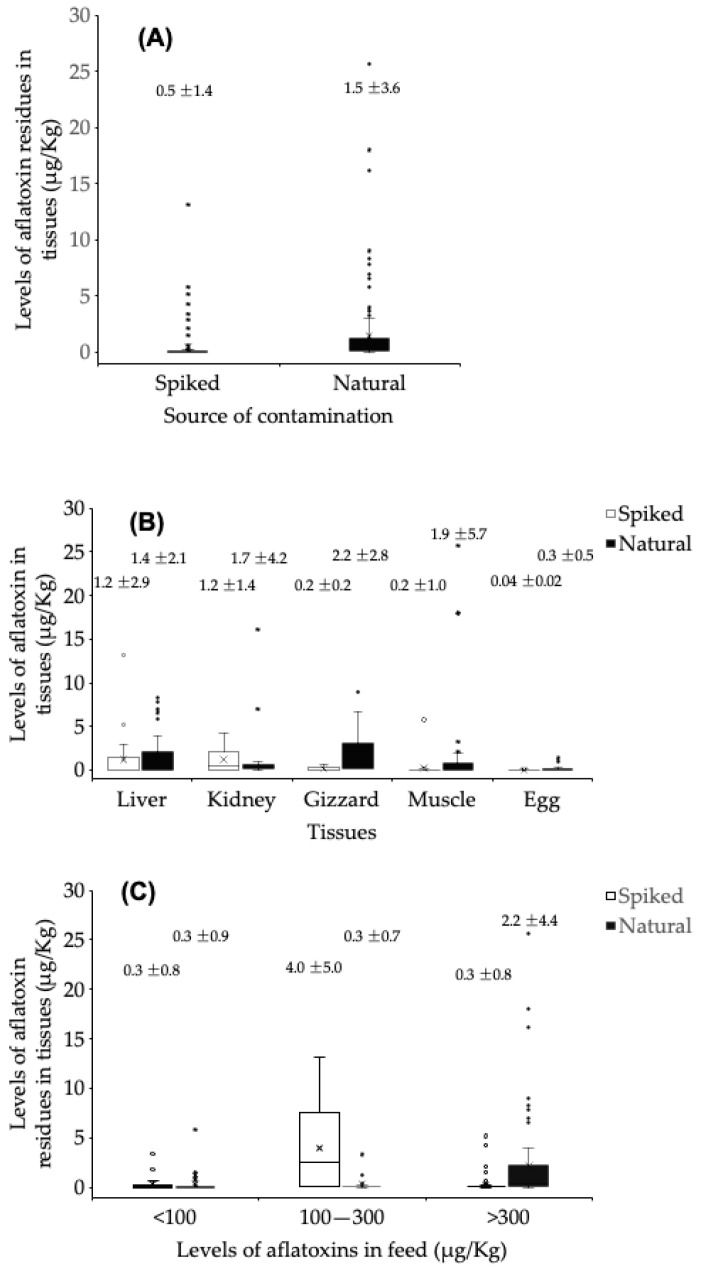
The effects of the source of aflatoxin contamination of feed—spiked versus natural contamination. Figures above each column are mean ± standard deviation. (**A**) Overall comparison of aflatoxin residuals in tissues from birds fed spiked or naturally contaminated feed. (**B**) Aflatoxin residue concentration in different tissues from birds fed spiked or naturally contaminated feed. (**C**) Overall aflatoxin residue concentration in various tissues of birds fed spiked or naturally contaminated feed stratified by the aflatoxin concentration level in the feed. Solid blocks in each column represent the 25th to 75th percentile range. “X” indicated the median value observed. Bullets are values > 75th percentile.

When feed was contaminated with naturally occurring aflatoxins, then metabolically active organs generally retained more aflatoxin than when the feed was contaminated with purified aflatoxin (Figure 5A). Aflatoxin residues (Figure 5B) in the liver and kidney were generally the same or higher when the birds were fed spiked feed. For gizzards and muscle, the naturally contaminated feed usually resulted in higher tissue residue values. Eggs generally had low aflatoxin residues regardless of the contamination source. There was insufficient resolvable data for skin, heart, and spleen for a meaningful analysis. The level of contamination in the feed could impact whether natural or spiked contamination was more important in determining the amount of aflatoxin in the tissue residue (Figure 5C). When the feed was contaminated with <100 µg/kg of aflatoxins, then the results were the same for both contamination methods. If the feed contamination level was 100–300 µg/kg, then the tissue residues were higher if the feed was spiked than if it was naturally contaminated. If the feed was contaminated with >300 µg/kg, then the results were reversed, and the aflatoxin residue was higher in birds fed with naturally contaminated feed. Thus, both the method of contaminating the feed and the level of contamination impact the amount of aflatoxin retained in a tissue, and not all tissues behave similarly.

## 3. Discussion

Aflatoxin contamination of animal feed raises concerns about animal welfare, productivity, and the impact of residual contamination on secondary consumers, particularly humans. We evaluated residue levels of aflatoxins in chicken products and their association with the feed the animals consume, with a focus on the safety of chicken products for human consumption.

Globally, the regulated limit of total aflatoxin in food ranges from 4 to 30 μg/kg [40], with a median regulatory limit of 10 μg/kg [41]. In the European Union (EU), the limits for aflatoxin B_1_ and total aflatoxins are 2 μg/kg and 4 μg/kg, respectively [42], while the US allows up to 20 μg/kg [43,44,45]. Very few countries have a zero-tolerance policy for aflatoxins because it is virtually impossible to eradicate them. We found that when chickens were fed feed contaminated with aflatoxins ranging from 0.1 to 6400 µg/kg for 7–180 days, <1% of the aflatoxin in the feed is retained as residues in edible tissues despite the significant positive correlation between the concentration of aflatoxin in chicken feeds and the residue levels found in chicken tissues. The FDA set 300 μg/kg of aflatoxin as the maximum level allowed in corn used as an ingredient in chicken feed, although the maximum level allowed in the finished feed is 20 µg/kg [45]. Results from trials in which chickens were fed feed contaminated with >300 μg/kg of aflatoxins resulted in <10 μg/kg of aflatoxin in tissues, which implies that grain contaminated with more than 300 μg/kg can still be used as chicken feed with only minimal risk to humans who consume the birds that ate the contaminated grain.

Reported residue accumulation levels >20 μg/kg FDA regulatory limit were found only in the muscle. Of the trials that reported this level of aflatoxin in the muscle, only 4% of the trials in which chickens were fed feed contaminated with >300 μg/kg of aflatoxin. In the majority of these trials (87/91), <5 μg/kg of aflatoxin was retained in the muscle. In most trials with other edible tissues, e.g., the liver, eggs, kidneys, gizzards, skin, heart, and spleen, all retained <1 μg/kg of aflatoxin. These data suggest that products derived from birds fed feed contaminated with aflatoxin levels exceeding current regulatory levels could still be allowed in the human food chain with only minimal health risks to humans. Note that birds consuming feed that was naturally contaminated, as would occur in a real-world setting, generally had higher aflatoxin residues than those that consumed artificially spiked feed. The average contamination level for all tissues, except eggs, from these birds was around the 2.0 µg/kg level.

In essence, chickens reduce human exposure to aflatoxins when contaminated grain is fed to the chickens rather than being used directly as human food. This relationship is both important and practical since 60–70% of commercial chicken feed usually is grain [46]. If finishing chickens can tolerate up to 300 μg/kg of aflatoxin in chicken feed, then grain containing even higher levels might also be safe for use in chicken feed if the birds can tolerate these levels of contamination without reducing their health and productivity. Using such highly contaminated grain as chicken feed would provide a use for grain that now is often destroyed or allocated to other uses with lower economic values such as ethanol production. This use of contaminated grain as chicken feed could also help increase food security in many developing countries where decreasing aflatoxin in human food and increasing chicken production are both important goals.

Increasing the amount of aflatoxin allowed in chicken feed must consider the impact of these increased levels on animal health and performance. The results from available studies of high levels of aflatoxins on chicken health and performance are inconsistent. One study reported low performance by chickens fed grain containing >500 μg/kg [47] of aflatoxin. A second study found that the productivity of birds fed grain containing 250–1000 µg/kg of aflatoxin B_1_ did not significantly differ from that of control birds and that even a dietary aflatoxin B_1_ concentration of 24,000 μg/kg in the feed was not lethal [48]. Therefore, further research is necessary to determine the levels of aflatoxin in chicken feed that do not affect animal health and performance and that result in tissue residue levels that are safe for human consumption.

Another consideration is the length of exposure of chickens to aflatoxin in the diet. The duration of exposure to aflatoxin in the feed influences the amount of toxin accumulated in edible tissues (Figure 3). Chen et al. [17] reported that aflatoxin was cleared from chicken tissues within four days when birds were switched to an aflatoxin-free feed. This rapid clearance is consistent with short-term exposure, but longer exposure can lead to increased accumulation. However, diets containing 200 µg/kg and 300 µg/kg of aflatoxin resulted in less than 5 µg/kg and 10 µg/kg of toxin residue, respectively. Also, exposure periods of 200 to 300 days far exceed the typical lifespan of commercial broilers, which usually are slaughtered at 35–50 days of age. This short life span within the commercial production cycle means that even prolonged exposure to relatively highly contaminated feed may result in only minimal toxin retention, and that withdrawal from contaminated feed shortly before slaughter could be an effective strategy for reducing residues. Even in developing countries where chickens may live longer and eat highly contaminated feed, the amount of aflatoxin retained in the tissues will usually be <20 µg/kg, which is below levels considered hazardous for human consumption.

Beyond the inherent aflatoxin-buffering capacity of chickens, dietary supplementation with various additives can further reduce tissue aflatoxin residues, enhancing food safety. In broilers fed 1 mg aflatoxin B_1_/kg, curcumin and nano-curcumin reduced liver residues from 10.8 μg/kg to 2.3 μg/kg and 4.65 μg/kg, and muscle residues from 4.7 μg/kg to 1.0 μg/kg and 2.0 μg/kg, respectively [49]. At 250 µg aflatoxin B_1_/kg in the feed, Toxfin and Novasil (0.30%) reduced liver residues from 2.9 μg/kg to 0.55 μg/kg and 1.2 μg/kg, and kidney residues from 0.9 μg/kg to 0.3 μg/kg and 0.4 μg/kg [50]. With 1 mg/kg of spiked aflatoxin B_1_ in the feed, silymarin (0.6 g/kg), *Spirulina platensis* (1 g/kg), and their combination reduced aflatoxin liver residues to 0.1 µg/kg, 0.1 µg/kg, and non-detectable levels, respectively; all also yielded non-detectable residues in muscle versus 1.1 µg/kg in the controls [51]. In birds fed 485–520 µg/kg of aflatoxin B_1_ in the feed, chelated trace minerals lowered liver residues from 1.6 μg/kg to 0.8 μg/kg and kidney residues from 3.2 μg/kg to 1.4 μg/kg [52]. At the EU limit of 20 μg/kg aflatoxin B_1_ in the feed [42], turmeric powder (400 mg/kg) reduced liver residues to non-detectable levels compared to 46 µg/kg in the controls [53]. Similarly, other binders and detoxification systems including bentonite [54], multicomponent mycotoxin detoxifying agent [55], and a novel mycotoxin detoxification compound [32] have similar potential to reduce aflatoxin residues in various chicken tissues. These findings collectively affirm that these potential feed additives can synergize with the chicken’s endogenous detoxification mechanisms, significantly reducing aflatoxin bioaccumulation even under high-exposure conditions. These additives should also improve the health of the birds that consume these contaminated feeds.

## 4. Conclusions

There is general consensus across all 33 studies that aflatoxin residues in tissues are much lower, i.e., <1%, than the aflatoxin contamination levels found in the feed in spite of variation in location where studies were conducted, the type of bird used, and the methodology used to contaminate the feed and to detect aflatoxin residues. Aflatoxin residues in chicken products were generally low, suggesting limited risk to human health from eating chicken. The ability of chickens to act as a “biological filter” to remove aflatoxins from the food supply provides a potential alternate use for contaminated grain. In particular, it reduces concerns that chicken consumption in less-developed countries poses a health risk due to aflatoxin contamination, even if the chickens are consuming relatively highly contaminated grain. Including additional additives in the feed has the potential to reduce these residue levels even further. The length of time of consumption of contaminated grain by the chickens is estimated to account for about 33% of the variation observed, and there is a significant chance that birds consuming contaminated feed for a long time, >100 days (Figure 3), will have higher aflatoxin residues in their consumable tissues. These conclusions indicate that further research to establish the maximum tolerable level of aflatoxin in chicken feed is warranted, as is the evaluation of other birds, such as ducks, geese, and turkeys, to determine if they too could serve as “biological filters” for aflatoxins in the food supply.

## 5. Materials and Methods

### 5.1. Data Collection and Curation

We systematically searched the Web of Science (Clarivate, London, UK) and PubMed (National Library of Medicine, National Institutes of Health, Bethesda, MD, USA) databases on 5 October 2023 using the following search phrase: ((AF OR AFB1 OR AFB2 OR AFG1 OR AFG2 OR AFL) AND (egg OR meat OR liver OR muscle OR gizzard OR organ OR kidney OR tissue OR residue) AND (poultry OR chicken OR layer OR broiler OR hen) AND feed). This search yielded 1887 articles, with the number reduced to 1523 when duplicates were excluded (Figure 1). Titles and abstracts were screened and 1470 articles were excluded because (i) the study did not involve broiler or layer chickens; (ii) the study did not measure both aflatoxins in the feed and residue in the tissues; (iii) the study was reported in a language other than English and could not be translated by Google translate; (iv) the study was outside the scope of this study, e.g., general and systematic reviews, book chapters, encyclopedias, case reports, and letters to the editor. We sourced, read, and critically analyzed the full texts of the remaining 53 articles, and retained 33 for further evaluation. These 33 articles all: (i) described peer-reviewed experimental research studies in which chickens (any age) were fed aflatoxin-contaminated feed, (ii) included data on aflatoxin residues in various tissues from chickens fed the aflatoxin-contaminated feed, and 3) specified the treatment protocols used.

Two independent reviewers extracted data by using a common data collection template and later resolved disagreements and discrepancies. A third reviewer verified the extracted data and confirmed whether the extraction protocol had been properly implemented. Information was collected, including the types of chickens used in the studies (i.e., broiler and layers), the concentration of aflatoxins in the feeds, the duration of feeding with the contaminated feed, and the concentration of aflatoxin residues or metabolites in multiple tissues after the chickens were sacrificed.

### 5.2. Data Quality Assessment

Two reviewers independently assessed the quality of the included studies. The potential risk of bias in the studies included in the meta-analysis was evaluated via the SYRCLE risk of bias tool for animal studies [56]. The assessment points were related to sequence generation, baseline characteristics, allocation concealment, random housing, blinding, random outcome assessment, incomplete outcome data, selective outcome reporting, and other potential sources of bias.

### 5.3. Data Analysis

All data analyses were performed using JMP Pro version 16.0.0 (SAS Institute, Cary, NC, USA). The correlation between the concentration of aflatoxins in chicken feed, the duration of consumption of the contaminated feed, and aflatoxin residues in chickens was determined by using Pearson’s correlation test. Some of the studies reported non-detectable levels of aflatoxin residues in chicken tissues. In these cases, we divided the limit of detection for the diagnostic test used by two and used this adjusted result in the analysis. The accepted level of statistical significance was *p* < 0.05. To determine the carryover rate of aflatoxins from feed to chicken tissues, we calculated the percent fold change, i.e., the ratio of aflatoxin in tissue: aflatoxin in feed.

The US Food and Drug Administration (FDA) specifies the action levels for aflatoxin in chicken feeds or ingredients, such as cottonseed meal as 300 µg/kg and as 100 µg/kg for corn and peanut products [45]. We categorized the aflatoxin levels in the chicken feeds into three categories and the subsequent residues in various tissues into five categories (Table 2).

## Figures and Tables

**Table 1:** Characteristics of the 33 included studies.

COA (µg/kg)	DOF (Days)	Country	Type	N	Tissues Evaluated	Diagnostic Test	Ref.
100, 200, 400	42	Pakistan	Broiler	120	Liver Muscle	HPLC	[10]
250	20	Saudi Arabia	Broiler	360	Liver	HPLC	[11]
300, 600	42	Pakistan	Broiler	180	Kidney Liver	ELISA	[12]
110, 117, 129, 345, 361, 385	21, 28, 35, 42	Saudi Arabia	Broiler	100	Gizzard Kidney Liver Muscle	HPLC	[13]
NR	NR	Iran	NR	50	Eggs Liver	HPLC	[6]
250	35	Egypt	Broiler	150	Liver Muscle	NR	[14]
3000	7	Japan	Broiler Layer	64	Liver Muscle	HPLC	[15]
50, 100	42	Japan	Layer Duck	270	Liver Muscle	HPLC	[16]
1300, 2100	35	USA	Broiler	48	Gizzard Muscle	NR	[17]
1,000	42	Spain	Broiler	120	Liver	HPLC	[18]
2500, 5000	32	Spain	Layer	80	Egg Muscle Liver Gizzard Kidney	TLC	[19]
600, 1200, 1800	7, 14, 21	USA	Broiler	336	Liver	TLC	[20]
5	21	Spain	Layer	72	Kidney Liver Muscle	HPLC	[21]
1600, 3200, 6400	7	Pakistan	Broiler	2,000	Liver Muscle	HPLC	[22]
123	42	China	Layer	336	Eggs	HPLC	[23]
5.8, 8.2	NR	Cameroon	Layer	48	Eggs Liver Muscle	ELISA	[24]
NR	NR	Pakistan	Broiler	264	Kidney Liver Muscle	HPLC	[9]
50	36, 64, 169	Italy	Broiler	163	Kidney Liver Muscle Skin	HPLC	[25]
2500	21	Columbia	Broiler	100	Kidney Liver	HPLC	[26]
100, 300, 500	54	Brazil	Layer	93	Eggs Muscle	TLC	[27]
NR	NR	United Arab Emirates	NR	NR	Eggs	LS-MS/MS	[7]
2500, 3900	280	India	Layer	144	Eggs Muscle	NR	[28]
100	22	Argentina	Broiler	25	Liver	HPLC	[29]
45	21	Serbia & Montenegro	Broiler	1,000	Liver	TLC	[30]
2500	28	Italy	Layer	96	Liver	EIA	[31]
NR	NR	Mozambique	Broiler	NR	Gizzard Liver	ELISA	[8]
100	42	Serbia	Broiler	96	Liver Muscle	LC-MS/MS	[32]
5000	28	China	Broiler	NR	Muscle	HPLC	[33]
1700, 3300	28	USA	Layer	16	Eggs	TLC	[34]
1700, 3300	28	USA	Layer	80	Heart Gizzard Kidney Liver Muscle Spleen	TLC	[35]
37, 69, 82, 130	21	China	Broiler	1,200	Eggs Liver	HPLC-MS/MS	[36]
0.1, 25, 52, 77	60	China	Broiler	180	Eggs Heart Liver Muscle	HPLC-MS/MS	[37]
47, 450	30	China	Broiler	75	Liver Muscle	ELISA	[38]

OA—Concentration of aflatoxin in feed; DOF—Duration of Feeding; ELISA—Enzyme-Linked Immunosorbent Assay; HPLC—High-Performance Liquid Chromatography; HPLC-MS/MS—HPLC–tandem mass spectrometry; LC—Liquid Chromatography; LC-MS/MS—LC–tandem mass spectrometry; TLC—Thin-layer chromatography; EIA—Enzyme Immunoassay; NR—Not reported.

## Data Availability

The raw data supporting the conclusions of this article will be made available by the authors on request.
